# Ethics of Future Disclosure of Individual Risk Information in a Genetic Cohort Study: A Survey of Donor Preferences

**DOI:** 10.2188/jea.JE2007425

**Published:** 2008-10-01

**Authors:** Kenji Matsui, Reidar K. Lie, Yoshikuni Kita, Hirotsugu Ueshima

**Affiliations:** 1 Department of Biomedical Ethics, the University of Tokyo; 2 Department of Health Science, Shiga University of Medical Science; 3 Center for International Health, the University of Bergen; 4 Department of Bioethics, the US National Institutes of Health

**Keywords:** Feedback, Psychological, Genetics, Japan, Preferences, Ethics, Research

## Abstract

**Background:**

Although genetic epidemiologic research has added an element of individualization to epidemiologic research, there is neither agreement nor much discussion on whether donors of genetic samples should be offered an opportunity to receive individualized results regarding their genetic susceptibility to disease. Little is known regarding donors’ preferences for future disclosure of individual results. The purpose of this study is to investigate the actual preferences of such donors with regard to receiving individual results, to explore the factors related to their decision, and then to discuss ethical issues regarding the disclosure of results.

**Methods:**

Participants (n = 1857) of an ongoing Japanese population-based genetic cohort study in Takashima, Shiga, in 2003, were asked at entry about their preferences with regard being recontacted by researchers in the future and whether they wanted to receive reports on their individual genetic results if genetic problems relevant to their health are discovered for which efficacious interventions might be available.

**Results:**

Most of the donors wished to be recontacted and receive reports, but some did not want any reports. Those who were younger, former/current drinkers, or had at least 1 parent who had had cancer were more likely to want the results, while those who had at least 1 sibling with a medical history of cancer were less likely to want the results.

**Conclusion:**

We observed a high level of positive preference for future disclosure of individual genetic results, which is in line with the professional views on the ethics of this issue. A well-considered procedure for ascertaining donors’ preferences for receiving the results of the research is required from an ethical perspective.

## INTRODUCTION

After considerable discussion over the past decades, it now seems to be agreed upon in the epidemiologic research community that research participants should be given the aggregate results of the research in which they have participated after its completion.^1^^-^^3^ However, there is neither agreement nor much discussion in the community on whether participants of epidemiologic researches should be informed, on an individual basis, of the results of the analysis,^4^^,^^5^ although the Convention on Human Rights and Biomedicine in 1997 held by the Council of Europe clearly stipulates that “everyone is entitled to know any information collected about his or her health.”^6^ On one hand, some epidemiologic studies may actually return several cross-sectional personal results to an individual participant at an immediate or intermediate stage. Moreover, it is nonetheless true in the practice of epidemiologic research that such individualized research results, especially those that can only be obtained through and after a decade-long follow-up, are rarely returned to individual participants, even when the individuals are traceable and the results relevant to the health of the individual in the future.^1^^,^^4^^,^^7^^,^^8^

Prompted by the launch of the Human Genome Project in 1990 and thanks to recent advances in molecular genetics, an increasing number of epidemiologists have initiated genetic epidemiologic research involving the collection and storage of human genetic samples. Along with follow-up data on the onset and course of the disease, epidemiologists are expecting to identify individuals who are genetically susceptible to a particular disease or at increased risk of the disease. It is hoped that such genetic epidemiologic research will ultimately be able to provide each individual with an increased number of tailor-made medical interventions for the treatment or prevention of the disease.^9^ Thus, epidemiologic studies have now entered a new epoch-making chapter of individualization by creating genetic information on an individual basis, which did not exist previously.

In 2006, following these trends in medical science and epidemiology, the recommendations of an ethics working group on Reporting Genetic Results in Research Studies held at the American National Heart, Lung, and Blood Institute were published. The report states that genetic test results obtained in research studies should be reported to the study participants when (1) the associated risk for the disease is significant; (2) the disease has important health implications to the participants; (3) proven therapeutic or preventive interventions are available; and (4) the tests have been performed in a laboratory of established analytical validity.^10^ Therefore, although the fourth criterion seems to be inapplicable to research outside the United States, even genetic epidemiologic studies, which are sometime population-based, should not be exempted as long as they meet at least the first 3 criteria. Since epidemiologists currently have genetic epidemiologic tools, it is highly likely that evidence of the predictive value of these genetic variants will accrue in the course of prospective epidemiologic studies and, therefore, that current epidemiologists who engage in genetics research may face growing pressure to return individual genetic findings that have an influence on disease risk.^10^ However, since the deliberations by the American National Heart, Lung, and Blood Institute lacked data on the views of actual participants who have donated their genetic samples to epidemiologic studies, it is still not certain whether the professional views reflected in these recommendations are consistent with the views of actual donors.

In the current study, we collected data on the preferences of genetic sample donors regarding future reports on individual genetic research results in an ongoing Japanese population-based genetic epidemiologic study. We also examined several potential factors that might influence these preferences. We conclude this paper by discussing the ethical issues concerning reporting of individual genetic risk information in epidemiologic research.

## METHODS

### Setting and Study Population

This study was conducted in 2003 as part of a prospective population-based genetic epidemiologic research in Japan-the Takashima study.^11^ The project conducted a baseline collection of various health-related information and biological samples, including genetic samples from residents in the towns of Takashima and Makino in Takashima County, Shiga, Japan, during May and June in 2003, in conjunction with the national annual health checkup program. In total, 2279 people attended the health checkup program in the area. The current study targeted the 1857 people (84.6% of the health checkup attendees) who consented at entry to donate their genetic samples to the associated genetic research in which analyses of the association between genetic profiles and onset of or outcome of lifestyle-related diseases such as cancer, stroke, cardiovascular disease, and dementia will be conducted in the future.

### Measures

**(1) Questions regarding Preferences for Future Individual Reports:** All the genetic analysis participants were asked to answer the following 2 short questions (Q1 and Q2) at the end of the consent form during the informed consent process: In case the researchers should discover at some time in the future that you have a particular genetic problem related to a serious disease(s) for which efficacious interventions might be available at that time, (Q1) “Would you wish to be recontacted by the researchers at that time so as to be given the opportunity to make a decision with regard to receiving the genetic risk information?” and (Q2) “What is your present preference with regard to knowing the details of such a genetic result in the future?” Q1 was provided so as to give each participant the opportunity to decline any future contact by the study group, because the researchers had reason to believe that some participants might not be interested in the information unless they had asked for it before.^12^ The options provided were “Yes, I wish to be recontacted” and “No, I do not wish to be recontacted.” Q2 was provided in order to make the individuals aware of the gravity of their choice and, secondly, to evaluate the percentage of individuals who actually want the information during the initial informed consent process in order to know their individual genetic risks that might be discovered at some time in the future during the course of the study. The options provided were “I wish to know the details,” “I do not wish to know the details,” and “I cannot decide my stance now.”

**(2) Variables of Sociodemographic Characteristics and Medical History Information:** Sociodemographic data and information regarding the medical history of the participants and their families were originally obtained through questionnaires associated with the Takashima Study. Through the review of a number of previous relevant studies in clinical or hypothetical settings-although they were not directly related to the actual setting of genetic epidemiologic research studies-on the opinion of patients and the general population with regard to learning the genetic test results in terms of personal genetic risk for diseases,^13^^-^^20^ we extracted several potential variables that could be used for the subsequent statistic analysis: sex (male = 1; reference, female = 2), age (y), educational history (y), religious status (following a religion = 1, no religion = 0; reference), marital status (ever-/currently married = 1, never married = 0; reference), number of children, number of grandchildren, household income (million yen), and personal and familial (father’s, mother’s, and sibling(s)’) medical history of diseases (yes = 1, no = 0; reference) such as hyperlipidemia (only with regard to the participant), hypertension, diabetes mellitus, stroke, cardiovascular disease, and cancer, and knowledge of the fact that certain diseases are inherited (yes = 1, no = 0; reference). We also used the data on the alcohol and smoking status (former/current drinker = 1, non-drinker = 0; reference and former/current smoker = 1, non-smoker = 0; reference) for the analysis because the researchers were convinced that these variables, which are related to people’s general perception of risky behaviors in their daily life, could also influence decision-making with regard to enrollment in a genetic analysis study.^21^^,^^22^

### Statistical Analysis

The chi-square test and univariate and multivariate logistic regression analyses were performed with SPSS^®^ 14.0J. Univariate logistic regression analysis was performed for assessing each possible variable that may correlate to the dependent variable, and then all the variables with a *P* value equal to or less than 0.10 were subsequently used in the multivariate logistic regression modeling with stepwise selection in order to explore factors that were likely to be associated with the participants’ preferences for future disclosure of individualized genetic risks. With regard to the dependent variable, those who wished for both future recontact and future reports on genetic risk information were confirmed as having “positive preference (= 1)” for receiving the information, and those who did not wish for either recontact or reporting were confirmed as having “negative preference (= 0; reference)”: all cases other than these 2 types were excluded from the analysis. Two-sided *P* values equal to or less than 0.05 were considered to be statistically significant.

## RESULTS

Three-quarters or a greater proportion of the participants provided their sociodemographic information to the researchers (Table 1). Two-thirds of the participants were females with a mean age of nearly 60 years. Most of the participants’ sociodemographic characteristics, such as education and household incomes, were consistent with (or similar to) those reported elsewhere.^23^^,^^24^ Moreover, most of the participants had the knowledge that some types of diseases are inheritable among blood relatives. More than 90% of the participants provided information regarding their medical history as well as those of their parents and siblings. According to the data (Table 2), prevalence of disease among the participants was similar to or slightly higher than that reported previously in the Shiga area.^24^

**Table 1.** Characteristics of the genetic analysis research participants of the Takashima Study, Shiga, Japan, in 2003

Characteristics		n (%)
Sex		(n = 1857; RR^‡^ 100%)
	Male	676 (36.4)
	Female	1181 (63.6)
Age, y		(n = 1857; RR 100%)
	Mean (SD)*	59.6 (14.1)
	Range	18-91
Marriage		(n = 1483; RR 79.9%)
	(Ever) Married	1436 (96.8)
	Never Married	47 (3.2)
Education, y		(n = 1449; RR 78.0%)
	≤6	62 (4.3)
	≤9	559 (38.6)
	≤12	463 (32.0)
	≤14	191 (13.2)
	≤16	172 (11.9)
	≤18	2 (0.1)
	Mean (SD)	11.3 (2.7)
Religion		(n = 1454 ; RR 78.3 %)
	Following a religion	1309 (90.0)
	No religion	145 (10.0)
Household income, million yen		(n = 1401; RR 75.4%)
	Mean (SD)	4.2 (2.9)
	Range	0-9 (0 = Do not know)
Number of siblings		(n = 1477; RR 79.5%)
	Median (QD)^†^	3.0 (1.5)
	Range	0-12
Number of children		(n = 1455; RR 78.4%)
	Median (QD)	2.0 (0.5)
	Range	0-8
Number of grandchildren		(n = 1380; RR 74.3%)
	Median (QD)	2.0 (2.0)
	Range	0-12
Alcohol status (% of former/current drinkers)		(n = 1840; RR 99.1%)
		821 (44.6)
Smoking status (% of former/current smokers)		(n = 1809; RR 97.4%)
		480 (26.5)
Knowledge of the inheritability of diseases (% of positive response)		(n = 1464; RR 78.8%)
		1416 (96.7)

*, standard deviation; †, quartile deviation; ‡, response rate.

**Table 2.** Personal and familial medical histories of the genetic analysis research participants

Medical history of disease (% with the disease)		n (%)	
Personal medical history			
	Hyperlipemia	204 (11.3)	(n = 1798; RR* 96.8%)
	Hypertension	356 (19.7)	(n = 1804; RR 97.1%)
	Diabetes mellitus	77 (4.3)	(n = 1785; RR 96.1%)
	Cardiovascular disease	51 (2.9)	(n = 1784; RR 96.1%)
	Stroke	32 (1.8)	(n = 1780; RR 95.9%)
	Cancer	39 (2.2)	(n = 1780; RR 95.9%)
Father’s medical history			
	Hypertension	255 (15.1)	(n = 1693; RR 91.2%)
	Diabetes mellitus	89 (5.3)	(n = 1680; RR 90.5%)
	Cardiovascular disease	154 (9.1)	(n = 1684; RR 90.7%)
	Stroke	163 (9.7)	(n = 1685; RR 90.7%)
	Cancer	287 (16.9)	(n = 1703; RR 91.7%)
Mother’s medical history			
	Hypertension	310 (18.2)	(n = 1702; RR 91.7%)
	Diabetes mellitus	99 (5.9)	(n = 1689; RR 91.0%)
	Cardiovascular disease	140 (8.3)	(n = 1682; RR 90.6%)
	Stroke	123 (7.3)	(n = 1687; RR 90.8%)
	Cancer	191 (11.3)	(n = 1692; RR 91.1%)
Sibling medical history			
	Hypertension	88 (5.3)	(n = 1673; RR 90.1%)
	Diabetes mellitus	77 (4.6)	(n = 1674; RR 90.1%)
	Cardiovascular disease	71 (4.2)	(n = 1677; RR 90.3%)
	Stroke	54 (3.2)	(n = 1666; RR 89.7%)
	Cancer	211 (12.5)	(n = 1684; RR 90.7%)

*, response rate.

Of the 1857 genetic research participants, 1845 (99.4%) and 1767 (95.2%) individuals answered Q1 and Q2, respectively, which means that 1758 (94.7%) people in total answered both (Table 3). Among these 1758 people, 1596 (90.8%) individuals wished to be recontacted if any genetic risk information of clinical relevance should be discovered, whereas 162 (9.2%) individuals did not wish for any future recontact. Of these 1596 individuals, 1517 also wanted specific disclosure of risk information and were thus considered to have a positive preference for this information, while 143 (88.2%) of these 162 individuals did not wish for any information and were thus considered to have a negative preference for the same.

**Table 3.** Participant preferences at entry for future recontact and future disclosure of individual genetic risk information

Questionnaire statement		n (%)			

“In case the researchers should discover at some time in the future that you have a particular genetic problem related to a serious disease(s) for which efficacious interventions might be available at that time,”	Preference	Overall (n = 1857)	Male (n = 676)	Female (n = 1181)	*P* value
(Q1)“Would you wish to be recontacted by the researchers at that time so as to be given the opportunity to make a decision with regard to receiving the genetic risk information?”	Yes, I wish to be recontacted. No, I do not wish to be recontacted. Answer missing	1599(86.1)	601(88.9)	998(84.5)	0.020
		246(13.2)	70(10.4)	176(14.9)	
		12(0.6)	5(0.7)	7(0.6)	

(Q2)“What is your present preference with regard to knowing the details of such a genetic result in the future?”	I wish to know the details. I do not wish to know the details. I cannot decide my stance now Answer missing	1521(81.9)	578(85.5)	943(79.8)	0.024
		162(8.7)	49(7.2)	113(9.6)	
		84(4.5)	24(3.6)	60(5.1)	
		90(4.8)	25(3.7)	65(5.5)	

Univariate logistic regression analysis showed an association between these preferences and the sex of the participants (odds ratio [OR] = 0.701; *P* = 0.061), age (for every 1-y increase, OR = 0.963; *P* < 0.001), education (for every 1-y increase, OR = 1.174; *P* < 0.001), religion (OR = 0.327; *P* = 0.031), household income (for every 1-million yen increase, OR = 1.141; *P* = 0.001), number of grandchildren (OR = 0.869; *P* = 0.001), alcohol status (OR = 1.851; *P* = 0.001), parental medical history of cancer (OR = 1.867; *P* = 0.011), and sibling medical history of cancer (OR = 0.620; *P* = 0.051). These variables were then put into a multivariate logistic regression model of positive preference for individual genetic risk information: The regression indicated that the participants who were younger, had a higher household income, had a drinking habit, and had a parental history of cancer were more likely to have a positive preference for receiving individual genetic risk information, compared to those who did not show the abovementioned characteristics; in contrast, participants whose sibling(s) had a history of cancer were more likely to show a negative preference for the same (Table 4). The other variables obtained in the univariate modeling were rejected in the multivariate modeling.

**Table 4.** Logistic regression with stepwise selection for the positive preference of participants for receiving individual genetic risk information

Variable entered (*α* = 0.05)	Odds ratio (95% CI)	p value
Age (1 year incr.)	0.97 (0.95, 0.99)	0.003
Household income (1 mil. yen incr.)	1.14 (1.05, 1.24)	0.002
Alcohol status	2.08 (1.27, 3.42)	0.004
Parental medical history of cancer	1.88 (1.03, 3.44)	0.040
Sibling medical history of cancer	0.54 (0.30, 0.95)	0.032

Note: 1006 cases (60.6%) out of the 1660 cases were retained throughout the modeling with stepwise selection. CI, confidence interval

## DISCUSSION

This is a novel study on the preferences of genetic research participants at entry with regard to receiving the individualized results of genetic risks that are likely to appear in the course of the research. It involves a population-based series of actual research participants in an ongoing prospective genetic epidemiologic study in Japan. Secondly, this study identified several factors of importance that indicated individual decisions regarding future disclosure of genetic susceptibility to disease. We found that there was a high level of positive preferences among genetic sample donors for receiving individualized genetic results in the future should researchers discover any clinically significant genetic risk information for which there might be an effective treatment or prevention at the time. We also found that younger donors who have parent(s) with a history of cancer, or those with higher household incomes or a drinking habit were more likely to want these results. In contrast, donors who have sibling(s) with a history of cancer were less likely to want these results. Moreover, our study indicated that although stroke, cardiovascular disease, or dementia is medically diagnosed to be as severe as cancer, the family histories of these diseases did not influence our subjects’ preferences for receiving results. Finally, our study shows that the preferences among actual genetic sample donors for disclosure of the results were indeed in line with the views of the Working Group of the American National Heart, Lung, and Blood Institute.

Similar to our findings, a previous Swedish study using hypothetical scenarios reported a high positive preference among people for receiving individualized genetic research results.^25^ This study surveyed general attitudes toward tissue donation for hypothetical biobank-based research among the general population in the county of Västerbotten, where collection of blood or tissue samples for a biobank project had been conducted previously. The investigators found that 83.4% of their subjects were interested in knowing the results of any research that could provide them with information about a genetic predisposition to disease: among them, 54.9% stated that they would want to know the research results only if some effective treatment or prevention was available, and 28.5% stated that they would want the results under all circumstances. However, approximately 9.7% of the subjects said they would not want the results under any circumstances. Besides this, results that were similar to ours were also documented in other studies in clinical, non-research settings.^14^^,^^20^^,^^26^^,^^27^ For example, Vernon et al. reported that 10% of their hereditary nonpolyposis colorectal cancer patients who had given blood for genetic testing were not interested in learning their genetic test results.^20^ Keogh et al. also reported that younger patients with breast cancer, whose relatives underwent and received results of genetic testing for *BRCA1*/*2* mutations, had a significantly greater positive response to the invitation letter for genetic testing, and that a greater percentage of individuals with a first-degree family history had actually chosen to receive the results, compared to individuals without first-degree relatives having breast cancer.^26^ Therefore, our findings, as well as these findings, suggest that when conducting genetic analyses or testing, researchers need to be aware of the research subjects’ or patients’ backgrounds and their wishes with regard to the disclosure of individual genetic results.

We have shown that the actual preferences of genetic sample donors for feedback on individual genetic risk information were more or less in agreement with the professional views of the ethics related to this issue. On the basis of this finding as well as the principle of respect for the autonomy of participants, which is one of the four fundamental ethical principles in biomedical research,^28^ we recommend that researchers conducting genetic epidemiologic studies convey the research results regarding individual genetic risk to each participant when significant implications to his/her health are confirmed in the course of research. On the other hand, this may be problematic or difficult for the epidemiologic research community. Some may therefore oppose it, insisting that the intrinsic goal of epidemiologic studies is to yield group results, not individual ones, and thus conveying individualized results is unnecessary or useless;^29^ it may also be argued that offering premature results based on only statistical significance would merely cause unnecessary confusion or fear in the participants^30^^,^^31^ or that generating and providing individual-level results is too costly and may destroy the main purpose of the research.^32^ It may also be said that researchers do not have a responsibility to return individual results if, and as long as, they clearly explain this to the participants in the informed consent process and the participants have consented to the condition that the results of the proposed research will not be available.

It must be admitted that these arguments against individual-level results, especially practical ones regarding the cost of procedures, may seem persuasive to a certain degree. It could also be argued from a utilitarian perspective that if the public health benefit of the genetic epidemiologic research outweighed the cost of providing individual results, such individual disclosure would be unethical.^33^ This may be the case indeed when there are no effective interventions to ameliorate the relevant diseases. However, when a research participant is discovered to have a serious condition for which there is an effective intervention, it would be morally difficult to sustain such arguments, not only because not providing feedback might worsen the condition of the participant, but also because it will damage the public trust in, and therefore destroy the public support of, both the relevant research project and the medical research community in general.^33^ Such distrust of the project and the research community would especially be fatal to long-term studies such as genetic epidemiologic studies. Moreover, from the perspective of justice, which is another fundamental ethical principle in research,^28^ it would also be unacceptable to enroll subjects unless those who contribute to the research are able to obtain potentially lifesaving information from the database of the project.^34^

We definitely have no intention of claiming that genetic epidemiologic researchers ought to convey immediately the genetic research results of their studies to each participant. We acknowledge that the implications of some genetic findings may not be immediately apparent and may need long-term follow-up before being confirmed sufficient to disclose.^35^ However, at some point in the future, researchers are likely to identify associations between the participants’ genetic profiles and certain treatable or preventable diseases. Given this possibility, and also because that is one of the intrinsic goals of genetic epidemiologic studies, current epidemiologic researchers should develop a prospective plan to handle such situations.^36^ Our results therefore support the recommendation that researchers should prepare themselves sufficiently-by discussing and clarifying the nature and circumstances of conveying the individualized results, developing a protocol, deciding a budget for it, and setting the appropriate informed consent procedure for a study that appropriately includes future provision for the results-prior to research implementation.^37^^,^^38^

As Knoppers, the chairman of the International Ethics Committee of the Human Genome Project, and her colleagues appropriately insisted, the participant’s right not to know genetic results must also be secured during and following the course of the research.^39^ The Special Emphasis Panel on Opportunities and Obstacles to Genetic Research of the NHLBI Clinical Studies of the American National Heart, Lung, and Blood Institute has addressed this issue and asserts that “invasion of privacy by recontact and recruitment for more research participation if the individual had not previously agreed to being recontacted” could be harmful.^40^ The right not to know is thus very fragile and could be easily infringed on by any future attempt to recontact the donors to ascertain their preference at the time.^36^ In order to provide appropriate protection to the rights of the donors, therefore, a well-considered procedure for ascertaining and securing their preferences for future recontact as well as future disclosure of the results must be planned at the very beginning of the research.

This study has several limitations. First, this was a cross-sectional study that focused on preferences at entry. Therefore, the respondents may react differently from their initial preferences if researchers actually discover any genetic risks and recontact him/her in the future. Second, the sample was drawn from a relatively small and rural geographic area of Japan and predominantly consisted of females. Third, although our data suggested that those individuals who have/had a drinking habit were more likely to want information regarding their genetic predisposition to a disease, it might only represent a gender difference because of the disproportionate representation of the male participant population among the non-drinkers and drinkers. Fourth, we did not assess the wishes of the participant when no effective intervention was available. Considering all this, therefore, our results must be interpreted with these limitations in mind and, thus, further studies on this issue under such a condition are required.

In conclusion, our study demonstrates that actual research participants in a genetic epidemiologic research want reports regarding their genetic information in the case of serious conditions against which effective interventions are available. From an ethical perspective, it seems unacceptable that participants should be denied access to information that could be clinically relevant for ameliorating their health conditions. Prior to initiating a research project in this era of genetic epidemiology, researchers should therefore prepare a procedure for ascertaining participant preferences with regard to future disclosure of results and securing their rights to access relevant information and the ability to decline information if they prefer.
